# The αGal Epitope of the Histo-Blood Group Antigen Family Is a Ligand for Bovine Norovirus Newbury2 Expected to Prevent Cross-Species Transmission

**DOI:** 10.1371/journal.ppat.1000504

**Published:** 2009-07-03

**Authors:** Maha Zakhour, Nathalie Ruvoën-Clouet, Annie Charpilienne, Brigitte Langpap, Didier Poncet, Thomas Peters, Nicolai Bovin, Jacques Le Pendu

**Affiliations:** 1 INSERM, U892, Université de Nantes, Institut de Biologie, Nantes, France; 2 Ecole Nationale Vétérinaire de Nantes, Nantes, France; 3 INRA UMR 1157, CNRS UMR 2472, IFR 115, Gif sur Yvette, France; 4 University of Luebeck, Institute of Chemistry, Luebeck, Germany; 5 Shemyakin and Ovchinnikov Institute of Bioorganic Chemistry, Moscow, Russia; University of North Carolina, United States of America

## Abstract

Among *Caliciviridae*, the norovirus genus encompasses enteric viruses that infect humans as well as several animal species, causing gastroenteritis. Porcine strains are classified together with human strains within genogroup II, whilst bovine norovirus strains represent genogroup III. Various GI and GII human strains bind to carbohydrates of the histo-blood group family which may be shared among mammalian species. Genetic relatedness of human and animal strains as well as the presence of potentially shared ligands raises the possibility of norovirus cross-species transmission. In the present study, we identified a carbohydrate ligand for the prototype bovine norovirus strain Bo/Newbury2/76/UK (NB2). Attachment of virus-like particles (VLPs) of the NB2 strain to bovine gut tissue sections showed a complete match with the staining by reagents recognizing the Galα1,3 motif. Alpha-galactosidase treatment confirmed involvement of a terminal alpha-linked galactose. Specific binding of VLPs to the αGal epitope (Galα3Galβ4GlcNAcβ-R) was observed. The binding of Galα3GalαOMe to rNB2 VLPs was characterized at atomic resolution employing saturation transfer difference (STD) NMR experiments. Transfection of human cells with an α1,3galactosyltransferase cDNA allowed binding of NB2 VLPs, whilst inversely, attachment to porcine vascular endothelial cells was lost when the cells originated from an α1,3galactosyltransferase KO animal. The αGal epitope is expressed in all mammalian species with the exception of the Hominidaea family due to the inactivation of the α1,3galactosyltransferase gene (*GGTA1*). Accordingly, the NB2 carbohydrate ligand is absent from human tissues. Although expressed on porcine vascular endothelial cells, we observed that unlike in cows, it is not present on gut epithelial cells, suggesting that neither man nor pig could be infected by the NB2 bovine strain.

## Author Summary

Noroviruses are a major cause of gastroenteritis in humans and other mammals such as pigs and cows. Various human strains are known to bind complex sugar structures related to ABO blood groups. A single strain does not recognize all people owing to the individual variation in ABO-related antigens. Binding to these molecules is required for infection since individuals lacking the appropriate sugar structures are not infected by a given strain. We now report that a cow-specific strain binds very specifically to the so-called xenoantigen, a sugar motif resembling B blood group antigen, present at the surface of the small intestine of cows. This antigen is absent from all human tissues since the human gene encoding an enzyme required for its synthesis has been inactivated by mutations during evolution of the Hominidaea lineage. Although present in other mammals such as pigs, we observed that this sugar motif is not expressed at the right location to allow infection, that is, the surface of the small intestine. Thus, the cow virus should not infect humans or pigs. Its adaptation to cows would prevent transmission to other species living in close contact with cows such as man and pig.

## Introduction

Caliciviruses are small non enveloped viruses approximately 27–35 nm in diameter with a positive-sense single-stranded RNA genome of 7.4 to 8.3 kb in size. Based on genomic organization and genetic analysis, the *Caliciviridae* family is divided into five genera, norovirus, sapovirus, vesivirus, lagovirus and becovirus (or nabovirus), and a sixth genus “recovirus” has been recently proposed [1]. Animal caliciviruses are suspected and confirmed causes of a wide spectrum of diseases including gastroenteritis (pigs, calves, cats, dogs and chickens), vesicular lesions and reproductive failure (pigs and sea lions), respiratory infections (cats and cattle) and a fatal hemorrhagic disease (rabbits and hares) [2]. Human and animal caliciviruses associated with gastroenteritis belong to the norovirus and sapovirus genera. The genus norovirus (NoV) has been divided into five genogroups, genogroups I to V [3]. More recently, a classification including two additional genogroups (VI and VII) has been suggested [4]. Human strains are classified into genogroups I, II, IV, VI and VII. Analysis of the complete ORF2 sequences, encoding the capsid protein, of genogroups I and II demonstrates high diversity and at present, 8 clusters have been defined within genogroup I (GI-1 to GI-8) and 19 within genogroup II (GII-1 to GII-19). Porcine NoV have been classified into GII-11 and recently into two novel genotypes (GII-18 and GII-19) [5]. NoV are also detected in calves. The first bovine NoV strain, Bo/Newbury2/1976/UK, was isolated from calves with diarrhea in the United Kingdom [6]. Later, another distinct genotype of bovine NoV, Bo/jena/78/GER was identified in Germany [7]. They belong to genogroup III [8],[9],[10], in which the Jena virus and the Newbury2 (NB2) are respectively the prototype of genotypes GIII-1 and GIII-2. Two other enteric bovine caliciviruses have been described, the Newburry agent 1 in the UK [11] and Nebraska strain in the USA [12]. Thus, a fifth genus named becovirus (or nabovirus) includes these two bovine viruses since they present significant differences with the other genera of the *Caliciviridae* family [13].

Human NoVs (HuNoV) have been found to recognize histo-blood group antigens (HBGAs), with different strains showing distinct specificities [14],[15]. HBGAs are complex glycans present on many cell types including red blood cells and vascular endothelial cells, as well as on the epithelia of the gastrointestinal, uro-genital and respiratory tracts. They can also be present in a soluble form in biologic fluids such as saliva and milk. HBGAs are synthesized from a series of precursor structures by stepwise addition of monosaccharide units via a set of glycosyltransferases. According to the CAZY classification (http://www.cazy.org/), three glycosyltransferases families are involved in their biosynthesis. The GT11, GT10 and GT6 families which encode α1, 3/4fucosyltransferases, α1,2fucosyltransferases and enzymes related to the A and B enzymes of the ABO system, respectively. Some of the corresponding genes are polymorphic whilst others are expressed in a species-specific manner. In humans, the pleiotropic interaction of alleles at three loci, *FUT3*, *FUT2* and *ABO* determines the Lewis, Secretor, and ABO phenotypes, respectively [16]. The corresponding antigens are involved in HuNoV recognition of human digestive tissue and are required for infection [14],[15]. The GT6 gene family (*ABO* family) comprises three other members which encode α1,3galactosyl or N-acetylgalactosaminyltransferases. An α1,3galactosyltransferase acts on the type 2 precursor disaccharide (Galβ4GlcNAc) to give the αGal epitope, also called Galili antigen (Galα3Galβ4GlcNAc), which is expressed in all mammalian species except hominids since in humans, gorilla and chimpanzee the *GGTA1* (glycoprotein, alpha-galactosyltransferase 1) gene has been inactivated by several mutations and is therefore a pseudogene [17]. Another gene of the same family encodes a distinct α1,3galactosyltransferase which acts on the glycosphingolipid lactosylceramide to generate the isoglobotrihexosylceramide (iGb3: Galα3Galβ4Glcβ-Cer). The corresponding iGb3 synthase gene does not appear to be functional in humans, although this is actively debated [18],[19]. Finally, the last enzyme of the GT6 family known at present is an α1,3N-acetylgalactosaminyltransferase which acts on the glycosphingolipid called globoside (Gb4) or P blood group antigen to generate the Forsmann antigen (GalNAcα3GalNAcβ3Galα4Galβ4Glc-Cer). This enzyme, the Forsmann synthase, is not active in humans [20]. Recently, our group described other genes of the GT6 family (GT6m5 to GT6m8). However, they are not present in all vertebrate or mammalian genomes and the enzyme activity of the corresponding proteins have not been characterized as yet [21].

The genetic and antigenic relatedness of human and animal noroviruses suggests the possibility for inter-species transmission as illustrated by the recent detection of sequences close to GII-4 HuNoV in swine and cattle in Canada [22],[23]. Although animal NoVs have not yet been isolated from human, human infection with NoV related to genogroup III bovine NoV has been suggested by the presence of serum antibodies against bovine GIII-2 among veterinarians in the Netherlands [23]. Moreover the use of phylogenetically conserved cellular receptors appears as another risk factor for cross-species transmission. Attachment of the virus to a host ligand constitutes a first step of the viral infection process, and it has been observed that when the receptor is conserved between several species, these are more likely to be infected by viruses that use the shared receptor [24]. HBGAs can be conserved across high phylogenetic distances as shared epitopes have been found between bacteria, invertebrates, plants and mammal [25]. ABH-related structures have been characterized in the gut of all mammalian species tested so far [26]. The use of such molecules as primary ligands by HuNoV strains as well as by RHDV [27], a rabbit calicivirus of the lagovirus genus, prompted us to look for the ability of a bovine NoV to recognize HBGAs potentially present on bovine, as well as on porcine and human digestive epithelial cells. Such a shared ligand could help these viruses to propagate between the three species which live in close contact and at high densities in areas of intensive breeding.

## Results

### Recombinant NB2 VLPs bind to bovine digestive tract cells through a carbohydrate ligand distinct from A, B, H or Lewis histo-blood group antigens

**Figure 1:**
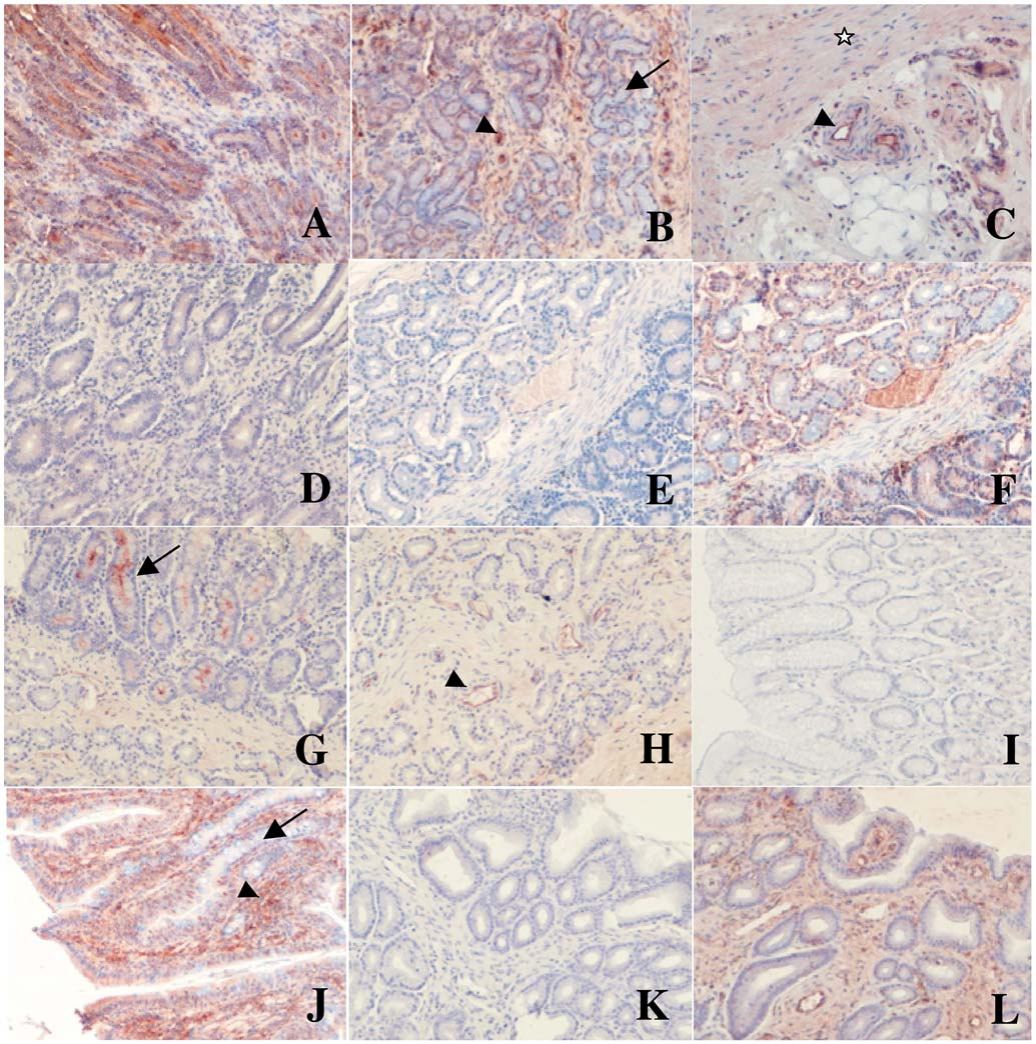
Analysis of rNB2 VLPs binding and of their ligand expression by immunohistology. VLPs at 1 µg/ml were incubated on tissue sections and detected as described in the Materials and Methods section. Binding of rNB2 VLPs to bovine duodenum surface epithelium (A), glands (B), smooth muscle (C) and negative control in absence of rNB2 VLPs (D). Effect of a 10 mM sodium periodate treatment prior to VLPs incubation (E) and serial control section pretreated in the same conditions without periodate (F). Staining of the surface epithelium (G) and vascular endothelium in connective tissue (H) of bovine duodenum by an anti-αGal mAb. Lack of binding of rNB2 VLPs on human duodenum (I). Binding of rNB2 VLPs to porcine duodenum (J). Effect of α–galactosidase treatment prior to rNB2 VLPs incubation on a porcine duodenum tissue section (K) and control section pretreated in the same conditions in absence of enzyme (L). Epithelial cells are indicated by arrows, vascular endothelium cells by small arrowheads, and smooth muscle cells by a star.

We previously demonstrated the binding of VLPs of caliciviruses to carbohydrate epitopes using immunohistochemistry as a starting method [27],[28]. We now used the same method in order to determine if the bovine NB2 strain of norovirus could similarly bind to a carbohydrate ligand expressed in the gut. Tissue sections prepared from the entire bovine gut were thus incubated with rNB2 VLPs and their binding was detected using antibodies. Specific staining, only visible in the presence of VLPs, was readily observed as shown on Fig. 1. In the duodenum, rNB2 VLPs attached to the epithelial cells of the crypts of Lieberkühn located at the surface of the mucosa (Fig. 1A), but not to the epithelial cells of the Brünner's glands which are located deeper in the mucosa (Fig. 1B). Unlike what was previously observed for other caliciviruses, binding of the VLPs was not restricted to epithelial cells. It was also observed on vascular endothelial cells and erythrocytes. These are clearly visible surrounding Brünner's glands (Fig. 1B) or in the serosa layer (Fig. 1C). In addition, other cell types such as smooth muscle cells were labelled, albeit less strongly. In order to determine if the binding involved recognition of carbohydrate epitopes, tissue sections were pretreated with sodium periodate prior to incubation with the VLPs since periodate oxidation cleaves C-C bonds with vicinal hydroxyl groups as found on sugars. Sialic acid residues are more sensitive to mild oxidation than other sugars. Since many viruses are known to use sialic acids as ligands [29], bovine duodenal tissue sections were first treated with 1 mM sodium periodate. This treatment did not affect the staining after incubation of the rNB2 VLPs (data not shown). Tissue sections were thus pretreated with 10 mM periodate. At that concentration, the staining was completely lost (Fig. 1E and 1F), suggesting that rNB2 VLPs recognize a neutral glycan structure expressed both on digestive surface epithelial cells and other cell types including vascular endothelial cells.

**Figure 2:**
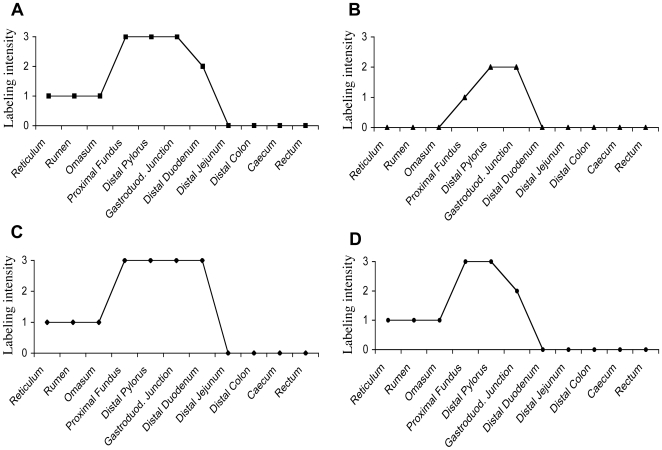
Expression of rNB2 VLPs ligands and of HBGAs along the bovine digestive tract. Expression of rNB2 VLPs epithelial ligands and HBGAs on the various parts of the digestive tract was determined by immunohistochemistry (see Fig. 1). Intensity of labeling shown on the y axis was visually graded from strongly positive (grade 3), moderately positive (grade 2) to weakly positive (grade 1) and negative (grade 0). (A) NB2 VLPs ligands ; (B) αGal epitope detected using either the GS1-B4 isolectin or the 4F102c2 mAb ; (C) A blood group antigen detected using mAb 9113D10 which recognizes all types of A epitopes ; (D) Htype2/Le^y^ epitopes detected using either the UEA-I lectin, or the 19-0LE mAb. *In addition to epithelial cells, rNB2 VLPs, GS1-B4 and the 4F102c2 mAb stained the vascular endothelium throughout the digestive tract.

Since other norovirus strains are known to bind to neutral carbohydrates of the histo-blood group family, we next sought to determine the expression of such epitopes throughout the bovine gut in order to relate it to the binding of rNV VLPs. To this aim, a set of antibodies as well as the UEA-I and GS1-B4 lectins were used and we observed that some, but not all, of these reagents clearly labeled bovine gut tissue sections. The A histo-blood group antigen was detected, but not the B antigen. Strong positivity was also observed using reagents that detect H type 2/LeY epitopes. In contrast, very little or no staining was detected using reagents specific for type 1 or type 3 based structures. Only the anti-Le^a^ antibodies stained scattered goblet cells in the duodenum and colon, whereas the anti-H type 1, anti-H type 3 and anti-Le^b^ did not give detectable specific labeling. Similar to that observed for rNB2 VLPs ligands, the A and H type 2/LeY expression was most intense in the pyloric and duodenal surface epithelia and gradually decreased from distal duodenum to disappear from the distal part of the digestive tract (Fig. 2). Yet, the expression of these fucosylated structures was observed on epithelial cells only and therefore did not match with that of the VLPs. In addition to A and H histo-blood group epitopes, the αGal epitope detected by either a mAb or the GS1-B4 lectin was detected in the bovine gut. The staining obtained with these reagents matched that of the VLPs since, as with the anti-A and H type 2/LeY, it was maximal in the gastro-duodenal area and absent from the distal part of the gut (Fig. 2), but more specifically, since the labeled cells and the relative intensities of labeling were the same as those observed following incubation with rNB2 VLPs as described above (Fig. 1G and 1H). The pattern of labeling obtained with the anti-αGal reagents was thus indistinguishable from that obtained with the VLPs, suggesting that the latter might recognize a carbohydrate structure related to the αGal epitope.

### Characterization of the Newbury2 recombinant VLPs ligand

**Figure 3:**
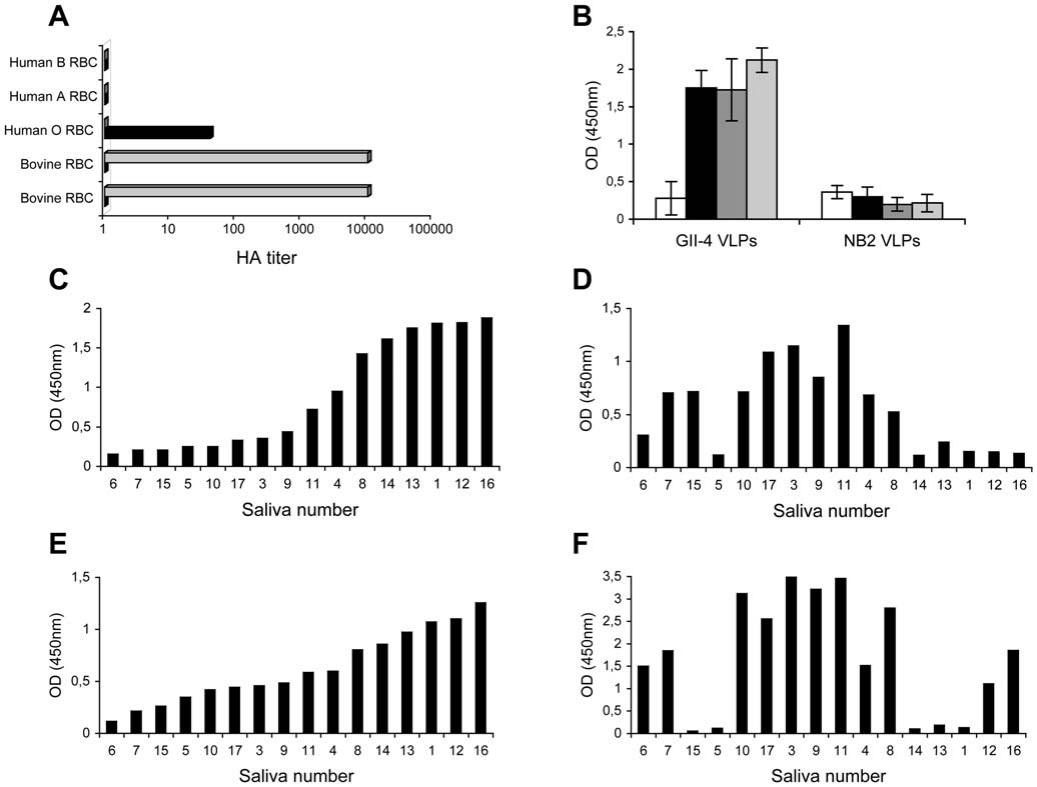
Red blood cell agglutination and saliva recognition. (A) Agglutination of bovine and human erythrocytes by VLPs from the bovine NB2 strain (gray bars) and from a human GII.4 strain of the Grimsby type which binds to Le^y^ (black bars). Erythrocytes from 2 individual cows and human erythrocytes from A, B or O blood group individuals were used. Results are shown as hemagglutination titers (HA titers). (B) Recognition of human saliva by VLPs from the bovine NB2 strain and the human Dijon (GII.4) strain determined by ELISA. Saliva samples were grouped into nonsecretors (white bars), A secretors (black bars), B secretors (dark gray bars) and O secretors (light gray bars) with 4 samples in each group. (C) Binding of rNB2 VLPs to individual bovine saliva samples determined by ELISA. Individual samples are numbered from 1 to 17 and ranked according to increasing OD 450 nm values. (D) Expression of the A blood group antigen in individual bovine saliva samples. (E) Expression of the αGal epitope detected by human natural anti-αGal antibodies in individual bovine saliva. (F) Expression of H/Le^y^ epitopes detected by the UEA-I lectin in individual bovine saliva samples. In D, E and F, samples are ranked as in C.

HBGAs were first characterized on human erythrocytes and it has been previously shown that hemagglutination (HA) can be used to define the HBGA specificity of human noroviruses [30]. We thus tested the ability of rNB2 VLPs to agglutinate bovine or human red blood cells. A strong agglutination of bovine erythrocytes was obtained both at 4°C and room temperature, but no agglutination at all was detected using human erythrocytes at either temperature, irrespective of their ABO phenotypes. In contrast, a GII.4 strain agglutinated human O blood group red blood cells, but not bovine erythrocytes (Fig. 3A). Since HBGAs are also present in saliva, we next assayed the binding of rNB2 VLPs to bovine and human saliva samples. When human saliva samples were assayed, no signal above background was obtained. In contrast, using the same amount of VLPs from a GII.4 strain, all human saliva samples from secretor individuals were strongly recognized, irrespective of their ABO phenotype. As previously observed for other human GII.4 strains, saliva samples from nonsecretor individuals were not recognized, showing specificity of the binding (Fig. 3B). On bovine saliva samples, rNB2 VLPs binding was readily detected, albeit with highly variable OD values, some samples giving a strong signal and others a signal at background level only (Fig. 3C). Nearly identical results were obtained with human natural anti-αGal antibodies (Fig. 3E). The binding of the UEA-I lectin and of anti-A and anti-B mAbs were assessed on the same set of samples. No binding of the anti-B was detected (data not shown). As depicted on Fig. 3D and 3F, the binding of the UEA-I lectin and of the anti-A mAb were heterogeneous too, but they were not related to each other, nor to that of the rNB2 VLPs. This indicates that individual bovine saliva samples are polymorphic with regard to the presence of either the A or H antigens. In addition, the lack of concordance between either the A or H antigens expression and the binding of rNB2 VLPs or human natural antibodies shows that individual differences in rNB2 VLPs binding were not due to non specific heterogeneity of the total amount of salivary glycans but were due to a true heterogeneity of the expression of the capsids ligand and of the αGal epitope. Taken together, these results clearly indicate that the ligand recognized by rNB2 VLPs is distinct from A, B or H antigens, but not from the αGal epitope and that it is not present on human red blood cells or in human saliva.

**Figure 4:**
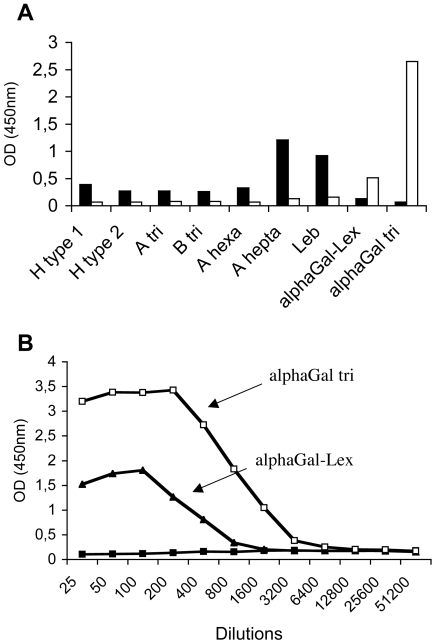
Binding of rNB2 VLPs to immobilized synthetic oligosaccharides. (A) A panel of neoglycoconjugates was coated on ELISA plates at 10 µg/ml and binding of either NB2 (white bars) or NV VLPs (black bars) was detected as described in the Materials and Methods section. OD 450 nm values on selected glycoconjugates are shown. No positive values were recorded for any of the other glycoconjugates listed in Table 1. (B) Binding of rNB2 VLPs to serially diluted structurally related PAA-conjugates. White squares: αGal trisaccharide ; black triangles: αGal-Lex ; black squares: mean of other PAA-conjugates (see Table 1 for structures). Results are shown as OD 450 nm as a function of the reciprocal of PAA-conjugate dilutions with 1/25 corresponding to 40 µg/ml.

In order to define the carbohydrate specificity of rNB2 VLPs, their ability to recognize a set of HBGAs related oligosaccharides was determined using an ELISA-based binding assay. The structure of all the oligosaccharides tested is given in Table 1. Binding was observed on two structures which share a common terminal galactose in α1,3 linkage, contrasting with the binding pattern of the human NV strain (Fig. 4A). None of the other tested oligosaccharides allowed binding above background. One of the αGal-terminated structures recognized by the bovine VLPs is fucosylated on the N-acetylglucosamine residue, generating a Lewis X epitope (αGal-Le x). The presence of this fucose residue partially impairs recognition as shown on Fig. 4B. Thus the preferred structure recognized by rNB2 VLPs among those tested is the trisaccharide Galα3Galβ4GlcNAc. Noticeably, the B blood group antigen which also presents a terminal galactose in α1,3 linkage was not recognized, consistent with the lack of agglutination of human B blood group erythrocytes and with the lack of binding to human saliva samples from B secretors.

**Table 1:** Neoglycoconjugates used to determine the carbohydrate specificity of rNB2 VLPs.

Trivial name	Oligosaccharide structure^a^
Tn	GalNAcα-R1
α–galactose monosaccharide	Galα-R1
α–fucose monosaccharide	Fucα-R1
A disaccharide	GalNAcα3Galβ-R1
B disaccharide	Galα3Galβ-R1
	Galα2Galβ-R1
	Galα6Glcβ-R1
Core 5	GalNAcα3GalNAcα-R1
H disaccharide	Fucα2Galβ-R1
Forsmann disaccharide	GalNAcα3GalNAcβ-R1
Core 8	Galα3GalNAcα-R1
Type 2 precursor	Galβ4GlcNAcβ-R1
Tαβ	Galα3GalNAcβ-R1
A trisaccharide	GalNAcα3(Fucα2)Galβ-R1, R2
B trisaccharide	Galα3(Fucα2)Galβ-R1, R2
H type 1	Fucα2Galβ3GlcNAcβ-R1
H type 2	Fucα2Galβ4GlcNAcβ-R1, R2
H type 3	Fucα2Galβ3GalNAcα-R1
αGal trisaccharide	Galα3Galβ4GlcNAcβ-R1
Gb3 (Pk)	Galα4Galβ4Glcβ-R1
iGb3	Galα3Galβ4Glcβ-R1
P1 trisaccharide	Galα4Galβ4GlcNAcβ-R1
Lewis a	Galβ3(Fucα4)GlcNAcβ-R1
Lewis x	Galβ4(Fucα3)GlcNAcβ-R1
3′-Sulfo-Lewis a	Su-O-3Galβ3(Fucα4)GlcNAcβ-R1
3′-Sulfo-Lewis x	Su-O-3Galβ4(Fucα3)GlcNAcβ-R1
A type 2	GalNAcα3(Fucα2)Galβ4GlcNAcβ-R1
B type 2	Galα3(Fucα2)Galβ4GlcNAcβ-R1
Lewis b	Fucα2Galβ3(Fucα4)GlcNAcβ-R1, R3
Lewis y	Fucα2Galβ4(Fucα3)GlcNAcβ-R1, R2
Sialyl-Lewis a	NeuAcα2,3Galβ3(Fucα4)GlcNAcβ-R1, R3
Sialyl-Lewis x	NeuAcα2,3Galβ3(Fucα4)GlcNAcβ-R1, R3
6-sulfo Sialyl-Lewis x	NeuAcα2,3Galβ3(Fucα4)(Su-O-6)GlcNAcβ-R1
Tk	GlcNAcβ3(GlcNAcβ6)GlcNAcβ3Galβ-R1
Lacto-N-tetraose (LNT)	Galβ3GlcNAcβ3Galβ4Glcβ-R3
Lacto-N-neotetraose (LNnT)	Galβ4GlcNAcβ3Galβ4Glcβ-R3
α-Gal-Lewis x	Galα3Galβ4(Fucα3)GlcNAcβ-R1
α-Gal pentasaccharide	Galα3Galβ4GlcNAcβGalβ4Glcβ-R1
Sialyl-Lewis x pentasaccharide	NeuAcα2,3Galβ3(Fucα4)GlcNAcβ3Galβ-R1
Sialyl-lacto-N-neotetraose (Sia-LNnT)	NeuAcα2,3Galβ4GlcNAcβ3Galβ4Glcβ-R1
Lactoneofucopentaose I (LNF I)	Fucα2Galβ3GlcNAcβ3Galβ4Glcβ-R1, R3
Lacto-N-fucopentaose II (LNF II)	Galβ3(Fucα4)GlcNAcβ3Galβ4Glcβ-R3
Lacto-N-fucopentaose III (LNF III)	Galβ4(Fucα3)GlcNAcβ3Galβ4Glcβ-R3
A hexasaccharide	GalNAcα3(Fucα2)Galβ3GlcNAcβ3Galβ4Glcβ-R3
A heptasaccharide	GalNAcα3(Fucα2)Galβ3(Fucα4)GlcNAcβ3Galβ4Glcβ-R3

a Oligosaccharides were used coupled to either polyacrylamide via an 3 carbon spacer (R1), or to human serum albumin via either a p-aminophenylethyl spacer (R2) or an acetyl phenylenediamine spacer (R3).

**Figure 5:**
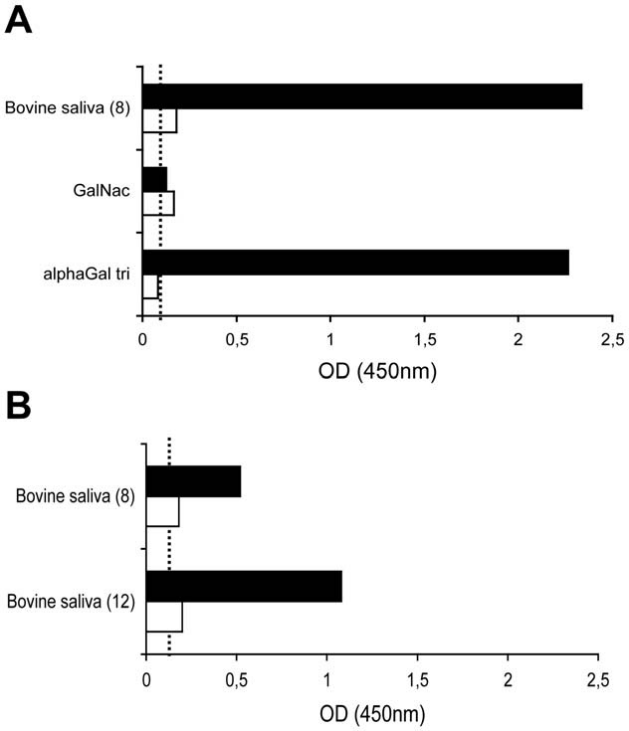
Inhibition of binding to bovine saliva by α-galactosidase treatment or by human natural anti-αGal antibodies. (A) Bovine saliva from a positive individual (#8) or PAA-conjugates (αGal trisaccharide and αGalNAc, see Table 1 for structures) were coated at a 1/1000 dilution or 10 µg/ml respectively and either treated with α–galactosidase from green coffee beans (white bars) or enzyme buffer only (black bars). (B) Bovine saliva from 2 positive individuals (#8 and #12) were coated as above and incubated with purified human anti-αGal (white bars) or blocking buffer only (black bars) for 2 hours prior to addition of rNB2 VLPs. Binding of VLPs was detected as described in the Materials and Methods section and shown as OD 450 nm values. Dashed lines indicate background level.

In order to determine if the ligand recognized in bovine saliva and digestive tissues corresponds to the αGal antigen, the effect of α–galactosidase treatment was tested. A saliva sample that allows good binding of rNB2 VLPs was chosen (sample # 8). The binding of VLPs was completely lost following the enzyme treatment. Activity of the enzyme was controlled using the Galα3Galβ4GlcNAc conjugate and binding to the treated conjugate was similarly lost after α–galactosidase treatment (Fig. 5A). The same treatment was applied to a bovine duodenal tissue section and staining completely disappeared (data not shown, see below for porcine tissues). In addition, human natural anti-αGal antibodies could inhibit the binding of rNB2 VLPs to bovine saliva samples (Fig. 5B). These results indicate that rNB2 VLPs specifically recognize the αGal antigen of the HBGAs family in bovine saliva and digestive tract.

### The binding epitope of Galα3GalβOMe at atomic resolution

**Figure 6:**
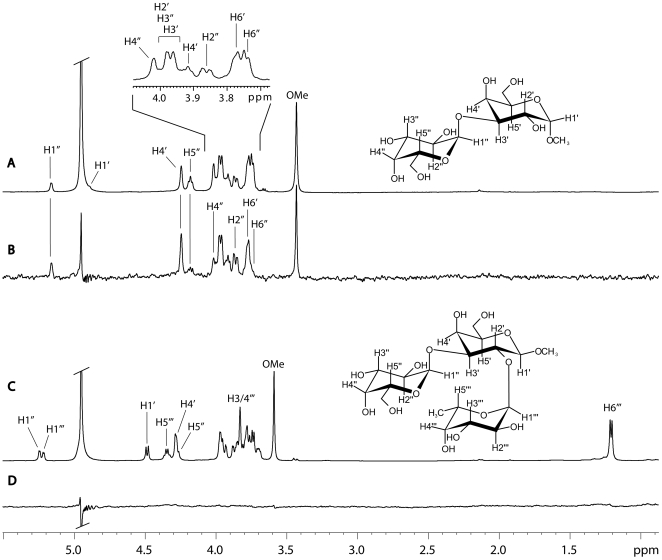
Observation of STD signals for the αGal disaccharide and the blood-group B trisaccharide. (A) Reference ^1^H NMR spectrum and (B) STD NMR spectrum of Galα3GalαOMe in the presence of bovine norovirus VLPs. (C) Reference ^1^H NMR spectrum and (D) STD NMR spectrum of the methyl glycoside of the blood group B-trisaccharide in the presence of bovine norovirus VLPs. STD signals are only observed for Galα3GalαOMe. The blood group B-trisaccharide shows no STD effects, and is therefore not recognized by the VLPs. Spectra were recorded at 500 MHz at a temperature of 282 K with 256 and 816 scans, respectively.

**Figure 7:**
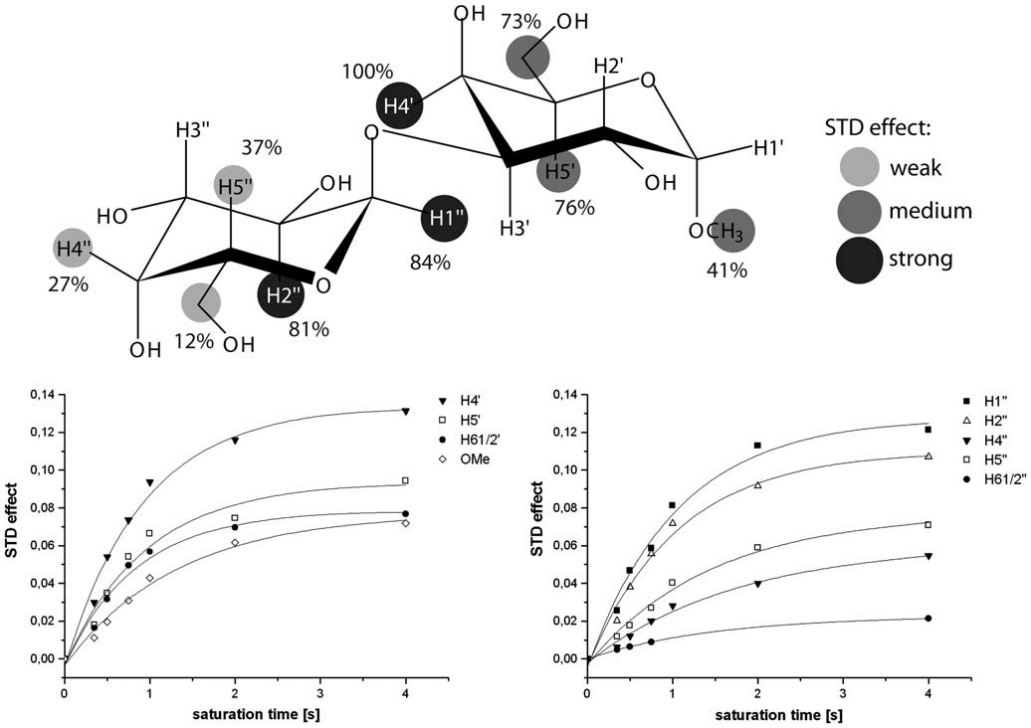
Binding epitope of Galα3GalαOMe binding to rNB2 VLPs (top) as derived from STD build-up curves (bottom). Greyscale circles encode the relative size of the saturation transfer and reflect the vicinity to protons in the binding pocket of rNB2 VLP. The larger the saturation transfer, the closer are the ligand protons to protons of the VLP binding pocket. From the Figure it is seen that the region around the glycosidic linkage is essential for recognition by the VLPs.

Binding of the Galili disaccharide (Galα3GalαOMe) to rNB2 VLPs was studied using saturation transfer difference (STD) NMR experiments [31]. This technique allows identification and characterization of ligand binding to large receptor proteins and yields binding epitopes of the ligand molecules at atomic resolution. It has been shown lately that the technique is well suitable for the investigation of carbohydrate receptor recognition by caliciviruses [32]. STD NMR spectra of Galα3GalαOMe and of the methyl glycoside of the blood group B trisaccharide in the presence of rNB2 VLPs at a saturation time of 2 s are shown in Fig. 6. The Galili disaccharide yielded sizable STD effects whereas no response at all was observed for the B-trisaccharide. This is in accordance with the biological assays that could not detect any binding to the B-antigen and underlines the strict specificity of the VLPs for the disaccharide moiety Galα3GalαOMe. STD NMR experiments of Galα3GalαOMe in the presence of rNB2 VLPs with increasing saturation times yielded STD build-up curves for individual protons. Fitting of these data to a single-exponential function (cf. Materials and Methods) delivered the binding epitope. It has been shown that strong spin-diffusion within large virus like particles requires the acquisition of build-up curves instead of single point measurements in order to generate reproducible binding epitopes. The binding epitope of Galα3GalαOMe derived from this analysis is shown in Fig. 7. It is remarkable that the protons around the glycosidic linkage received the largest fraction of saturation. This suggests that the α3-glycosidic linkage is in intimate contact with the VLP binding pocket. Obviously, the Galili disaccharide represents the central recognition element.

### Species specificity of the rNB2 VLPs ligand

The αGal is not expressed in apes due to inactivation of the *GGTA1* gene encoding the α1,3galactosyltransferase. Yet, it has been detected in all other mammalian species tested so far [17]. The lack of agglutination of human red blood cells and of binding to human saliva by rNB2 VLPs is consistent with the inability of humans to synthesize this antigen. In addition, no binding to the human duodenal mucosa was observed, confirming that rNB2 VLPs do not cross react with a human epitope present in the human gut (Fig. 1I).

Since pigs are known to be infected by noroviruses, and since they are known to express the αGal antigen, we tested the binding of rNB2 VLPs to the porcine gut by immunohistochemistry. A specific staining was readily observed throughout the digestive tract but surprisingly, it was restricted to non epithelial cells, including the vascular endothelium and to a lesser extent smooth muscle cells (Fig. 1J). Porcine duodenal tissue sections were then treated with the α–galactosidase from green coffee beans. Like on bovine tissue sections, the treatment completely abolished the binding of rNB2 VLPs to these cell types (Fig. 1K and 1L). The distribution of the αGal antigen in porcine digestive tract was then tested using an anti-αGal mAb and the GS1-B4 lectin. We observed that the two reagents gave stainings completely parallel with that obtained with the rNB2 VLPs (data not shown). This indicates that in porcine tissues, the αGal epitope is not expressed by digestive epithelial cells although it is present on other cells types recognized by the bovine viral capsids.

**Figure 8:**
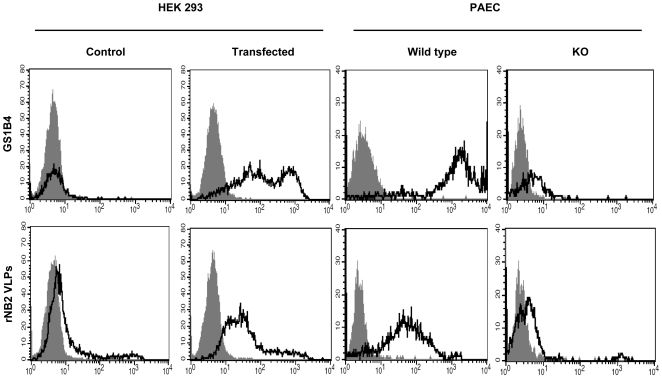
Cytofluorimetric analysis of the binding of rNB2 VLPs and the GS1-B4 lectin to rat Ggta1 transfected or control mock transfected HEK 293 cells and to porcine aortic endothelial vascular cells (PAEC) from wild type or Ggta1 KO pigs. Cells were incubated with either the GS1-B4 lectin or rNB2 VLPs and binding was detected as described in the materials and methods section. The respective negative controls (grey plots) correspond to either cells incubated in absence of the lectin or to cells incubated in the absence of VLPs. Another negative control performed in the presence of VLPs followed by incubation with an irrelevant immune rabbit antiserum and FITC-labeled anti-rabbit IgG yielded similar results. The log of fluorescence intensities in arbitrary units is plotted against the cell number.

Since the lack of αGal antigen in humans is due to inactivation of the *GGTA1* gene, we tested whether this event was sufficient to have caused the lack of recognition of human cells by rNB2 VLPs. Transfection of human HEK 293 cells with the functional rat *Ggta1* cDNA allowed expression of the αGal antigen as detected by flow cytometry using the GS1-B4 lectin. Control HEK 293 cells that lacked the αGal antigen were barely recognized by rNB2 VLPs. In contrast, a clear binding was observed on the *Ggta1* transfected human cells. Inversely, pig vascular endothelial cells spontaneously express the αGal antigen as documented from the labeling by the GS1-B4 lectin. The attachment of rNB2 VLPs to such cells was clearly detected by flow cytometry. However, we observed that pig vascular endothelial cells from a *Ggta1* KO pig, which accordingly are not stained by GS1-B4, were no longer recognized by the bovine VLPs (Fig. 8). These results confirm that rNB2 VLPs bind to the αGal antigen and that the expression of a functional α1,3galactosyltransferase is necessary and sufficient to allow their attachment to mammalian cells.

## Discussion

RNA viruses present a high risk of cross-species transmission since they are over-represented in the list of pathogens known to have crossed the species barrier [33]. This is most likely due to their particularly high mutation rate which allows them to evolve fast, providing rapid adaptation to a new host species. The use of phylogenetically conserved ligands facilitates the crossing of the species barrier. Since among Caliciviruses, human strains of norovirus and the rabbit hemorrhagic disease virus bind to HBGAs and since HBGAs can be phylogenetically conserved, we tested the possibility that the prototype of the bovine NoV, the Newbury2 strain classified in the GIII.2 cluster, could use such a conserved carbohydrate ligand to bind to bovine, porcine or human digestive epithelial cells. Our results demonstrated that VLPs from NB2 attach to the surface of the bovine duodenal epithelium by recognition of the αGal epitope of the HBGAs family and that this ligand cannot be used to infect either man or pig. This conclusion is based on the following observations: (1) the tissue distribution of rNB2 VLPs binding sites in the three species perfectly matched that of reagents specific for the αGal epitope which is absent from human and pig duodenal epithelial cells ; (2) among many immobilized HBGAs-related synthetic oligosaccharides, only those presenting the αGal epitope supported binding of the VLPs ; (3) α-galactosidase treatment of either tissue sections or bovine saliva completely impaired the binding of rNB2 VLPs ; (4) STD NMR experiments confirmed recognition of the αGal epitope at the atomic level ; (5) transfection of human cells with an α1,3galactosyltransferase cDNA allowed binding of rNB2 VLPs whilst inversely, their binding to porcine vascular endothelial cells was lost on cells from an α1,3galactosyltransferase KO pig.

The αGal epitope is structurally related to the histo-blood group antigen B type 2 since both share a terminal galactose in α1,3 linkage and the type 2 backbone structure (Galβ4GlcNAc). They only differ by the fucose residue of the B antigen, allowing some reagents such as some anti-B mAbs or the GS1-B4 isolectin to cross-react. Nevertheless, the possibility that rNB2 VLPs could recognize a B blood group epitope in addition to the αGal antigen is very unlikely since it failed to agglutinate human B blood group erythrocytes and to bind to human saliva from B blood group individuals of the secretor phenotype who strongly express B epitopes in their saliva. In addition, at the atomic level it was found by STD NMR experiments that the α3-glycosidic linkage serves as the central recognition element. Any disturbances close to this region such as the addition of a fucose residue in the 2-position of the reducing galactose moiety to yield the B-antigen would therefore impede with the binding process. One may speculate whether other positions that are more remote from the glycosidic linkage may be modified so as to obtain a better binder. Interestingly, we did not detect B blood group reactivity on cow tissue sections or saliva, making less likely the possibility for GIII.2 strains to evolve toward cross-recognition of the αGal and the B epitopes. Besides the species-specific expression of the αGal antigen on bovine digestive epithelial cells, another species-specific bovine characteristic was evidenced with regard to HBGAs expression. Indeed, we failed to detect HBGAs based on type 1 precursor (Galβ3GlcNAc) in bovine tissues or saliva, consistent with the results from earlier structural analyses of O-linked oligosaccharides from bovine salivary mucins which described the presence of type 2-based structures only [34],[35]. Those studies also failed to detect the αGal epitope. However, it could either be carried by N-linked glycans of salivary glycoproteins or the saliva studied could have originated from animals that did not express the epitope, consistent with our observation that not all bovine saliva samples could support attachment of rNB2 VLPs. Human NoV of either the GI or GII genogroups recognize HBGAs motifs based on both type 1 and type 2 precursors, but show stronger binding to type 1-based structures, particularly at 37°C [36],[37]. Human small intestine presents HBGAs based on type 1 as well as on type 2 backbones, suggesting adaptation of these strains to their host glycans. Even though cows can express A and H type 2 or Le^y^ antigens in their digestive tract, they may be less sensitive to infection by human NoV strains due to the lack of type 1-based structures. Interestingly, using saliva samples, we observed that similar to man and pig, cows are polymorphic regarding expression of the A and H antigens since some cows did not express either A, H or both. As these polymorphisms were unrelated to the αGal expression, the combination of the A, the H and the αGal polymorphisms is expected to generate eight subgroups of bovine and therefore significant individual variation which may be related to host-pathogens interactions.

Regardless, our results do not prove that the αGal ligand is necessary for infection of cows by GIII.2 strains, but several aspects support that possibility. Various human NoV strains that bind to HBGAs have been shown to infect their host in an HBGA-dependent manner [15]. Likewise, we recently obtained indirect evidence that the binding of RHDV, a lagovirus, to the H type 2 antigen is necessary for infection [38]. Thus, the conservation of the binding ability of an HBGA motif by the Newbury2 strain suggests that it may also be required for infection. Furthermore, histopathological analysis of the lesions of calves experimentally infected with NB2 indicated that they were restricted to the proximal small intestine [11], which coincides with the main site of expression of the αGal antigen on digestive epithelial cells. Finally, there is no clear evidence that bovine NoV can infect an other species [8]. Such viruses have never been detected in human or porcine samples, suggesting that they do not circulate in those species. This is to be expected if the αGal antigen serves as a receptor for infection. Nonetheless, one study described the presence of anti-GIII.2 antibodies in the serum of veterinarians in the Netherlands [23]. Bovine NoVs share cross-reactive epitopes with human NoVs [39],[40]. This cross-reactivity may explain the detection of anti-GIII.2 in some human serum samples.

In absence of cell culture models, it is very difficult to prove that a ligand is truly a receptor. The above described demonstrations that HBGAs can be compulsory ligands have been obtained through the analysis of the effect of their polymorphism on infection [41],[42],[43],[44]. Interestingly, we observed that the rNB2 VLPs salivary binding assay could distinguish between binder and non binder cows. If that polymorphism is also present at the level of digestive epithelial cells, it could be used to experimentally assay the sensitivity of either group to infection by the Newbury2 strain.

To date recombinants of bovine NoV and HuNoV have been identified and appear to be of frequent occurrence [4],[45],[46],[47],[48]. Cattle co-infection by a GIII.2 bovine strain and a human NoV could thus lead to the emergence of recombinant strains able to infect humans. However, this seems unlikely since as discussed above, the lack of type 1-based HBGAs in cow digestive epithelium may decrease recognition by HuNoVs, and since GI, GII and GIII strains are genetically distant and accordingly, inter-genogroup recombination, although recently observed, should be much less frequent than intra-genogroup recombination [4],[49].

Recent crystallographic analyses of the capsid protein domain of a GI.1 and a GII.4 NoV interacting with oligosaccharides showed that the two strains use distinct binding sites on the capsid protein protruding domain, although they bind to very similar oligosaccharides [50],[51]. A fucose residue is involved in both instances, although it is more essential to the GII.4 binding site than to the GI.1 binding site. Here we showed that the best binder of a GIII.2 strain is a related carbohydrate structure devoid of fucose and that addition of an α1,3-linked fucose to the backbone N-acetylglucosamine impaired recognition. It will thus be interesting to define the mode of recognition of the αGal trisaccharide by the NB2 strain in order to know if its binding site corresponds to one of those already characterized for either GI or GII strains. This knowledge should provide crucial information to understand how NoVs adapt to their host species and evolve to maintain recognition of diverse HBGAs that allow binding to allotypic and/or xenotypic host molecules.

The Galili antigen has mainly been studied in the context of xenotransplantation since it was originally observed that humans naturally produce antibodies against it and since these antibodies were shown to be the primary cause of hyperacute rejection of pig xenografts organs in human and hominids [17]. But the reason why the *GGTA1* gene has been inactivated in the Hominidaea lineage an estimated ∼28 MA ago is unclear. The loss of the αGal epitope allows the generation of so-called natural antibodies, similar to the generation of anti-A or anti-B natural antibodies in the ABO allogenic system, probably because some bacteria express identical or cross-reactive epitopes. These anti-αGal antibodies can recognize pathogens that carry the xenogenic epitope. Thus, envelopped viruses produced in animal cells that express a functional α1,3galactosyltransferase carry the αGal antigen on their envelope glycoproteins and the presence of natural antibodies directed against this epitope in human serum leads to their rapid elimination [52],[53]. For this reason it has been proposed that inactivation of the *GGTA1* gene in the Hominidaea lineage may have allowed escape from a highly pathogenic virus thanks to the natural anti-carbohydrate antibodies [17]. Our observation that an animal pathogen can use the αGal antigen as a ligand additionally suggests that the loss of GGTA1 may have allowed escape from some NoV strains. Clearly, at present GIII NoVs are of moderate or low pathogenicity. Nevertheless virulence evolves and past NoVs may have been much more virulent than present ones. The loss of the αGal ligand may have contributed to escape dreadful past NoV epidemics in hominids. Alternatively, the loss of the GGTA1 enzyme may have been completely independent from NoV infection. In this case, NoVs would have more recently evolved carbohydrate-binding specificities adapted to a broad spectrum of mammalian species. Regardless, the exquisite specificity of the NB2 strain for the αGal epitope is well adapted to its bovine host, but inversely should restrict its possibilities of cross-species transmission.

## Materials and Methods

### Ethics statement

All animals were handled in strict accordance with good animal practice as defined by the relevant national and/or local animal welfare bodies.

### Reagents

The BG-4 anti-H type 1 specific monoclonal antibody (mAb) was purchased from Signet laboratories (Dedham, CA) and mAbs 19-OLE, 7-LE and 2-25LE were obtained from Dr. J. Bara (CNRS, Villejuif, France). They are an anti-H type 2 showing a slight cross-reactivity with Le^y^ (unpublished results), an anti-Le^a^ and an anti-Le^b^, respectively. The anti-H types 3 and 4 mAb MBr1 was purchased from Covalab (Villeurbanne, France). The anti-A (all types) mAb 9113D10 was obtained from Diagast (Loos, France). The anti-B mAb ED3 was a kind gift from Dr. A. Martin (CRTS, Rennes, France). The anti-αGal 4F102c2 was a kind gift from Dr. A. Bendelac (Howard Hughes Medical Institute, Chicago, IL). The lectin from *Griffonia simplicifolia* B4 isolectin 1 (GS1-B4), either peroxidase or fluorescein isothiocyanate (FITC) conjugated, which recognizes α1,3-linked terminal galactosyl residues, was purchased from EY Laboratories (San Mateo, CA USA) and Vector Laboratories (Burlingame, CA USA), respectively. The lectin from *Ulex europaeus* (UEA-1) peroxidase conjugated, which recognizes H type 2 and Le^y^ was obtained from Sigma (St Louis, MO). Alpha-galactosidase from green coffee beans was purchased from Sigma. The anti-GII and anti-GIII rabbit polyclonal antisera were prepared at the Veterinary School of Nantes by immunizing rabbits with VLPs from GII.4 (Dijon 171/96) and GIII.2 (NB2) strains, respectively.

Synthetic oligosaccharides as polyacrylamide conjugates were prepared as described previously [54],[55]. Oligosaccharides coupled to HSA (human serum albumin) were obtained from IsoSep AB (Tulligen, Sweden). The structure of all oligosaccharides used is given on Table 1. The disaccharide Galα3GalαOMe was obtained from Calbiochem. The methyl glycoside of the B antigen has been synthesized enzymatically [56].

### Preparation of VLPs

The recombinant virus-like particles were prepared by infecting High-five insect cells with recombinant baculoviruses according to a previously described method [57]. The Dijon GII.4 171/96 strain [58] and the GI.1 Norwalk strain (NV) constructs were kind gifts of Dr. E. Kohli (University of Burgundi, Dijon, France) and Dr. X. Jiang (Cincinnati Children's Hospital Medical Center, Cincinnati, Ohio, USA), respectively. Five days post-infection High-Five lysed cells and media were centrifuged at 4500 rpm for 15 min and the supernatants collected and centrifuged at 25, 000 rpm for 3h30 in a SW28 rotor. The pellets were resuspended in distilled water and submitted to 2 rounds of purification on a sucrose gradient. The gene encoding the capsid protein of the NB2 norovirus cloned into pFastBac vector (InVitrogen) was a kind gift of Drs S. Oliver and J. Bridger (Royal Veterinary College London). Competent E. coli DH10BAC cells, containing baculovirus shuttle vector plasmid were used to generate recombinant bacmids according to the manufacturer's instructions (Invitrogen). Bacmids were introduced into *Spodoptera frugiperda 9* (Sf9) insect cells by lipofection and recombinant baculovirus were recovered. NB2 VLPs were produced by infection of *Spodoptera frugiperda 9* (Sf9) insect cells at a MOI∼5 PFU/cell. VLPs were purified as described previously [59] by double CsCl density gradient centrifugation. The composition of the VLPs was confirmed by polyacrylamide gel electrophoresis with Coomassie blue staining and VLP integrity was monitored by negative stain electron microscopy using 1% uranyl acetate stain. VLPs were stored in CsCl at 4°C.

### Tissue samples and immunohistochemical analysis

Bovine and porcine tissues samples from the oesophagus to the rectum were obtained from healthy animals autopsied at the National Veterinary School of Nantes. Human gastroduodenal junction samples had been obtained from organ donors before the law 88–1138 of December 20, 1988 concerning resection of human tissues after death for scientific investigations. Animal tissues were fixed in formalin and human tissues were fixed in ethanol 95% for 48 hours, and paraffin embedded. Sections (5 µm) were rehydrated in graded ethanol and washed in phosphate-buffered saline (PBS). Endogenous peroxidase was inhibited by using methanol/H_2_O_2_ 0.3% for 20 minutes. Sections were then washed in PBS for 5 minutes and covered with PBS/bovine serum albumin (BSA) 1% for 30 minutes at room temperature in a humid atmosphere. After washing in PBS, sections were covered with either the primary antibodies (HBGAs mAbs), with the peroxidase-conjugated GS1-B4 or UEA-I lectins at 10 µg/mL, or with rNB2 VLPs at 1 µg/ml, diluted in PBS/BSA 1% and left at 4°C overnight. Sections were then rinsed 3 times with PBS and incubated with either biotinylated anti-mouse immunoglobulin IgG (Vector Labs, Burlingame, CA) or with rabbit anti-NB2. After washing in PBS, the sections were covered with either peroxidase-conjugated avidin (Vector laboratories) or with peroxidase conjugated anti-rabbit IgG (Uptima, Montluçon, France) for 45 minutes. Reactions were revealed with 3-amino-9-ethylcarbazol, and counterstaining was performed with Mayer's hemalun.

Periodate treatment was performed immediately after the endogenous peroxidase quenching step by incubating sections with either 1 mM or 10 mM sodium periodate in 50 mM sodium acetate buffer, pH 5.0, for 30 minutes at room temperature, followed by a 10 minutes incubation with 1% glycine in PBS. Control sections were treated similarly with the same buffer but without sodium periodate. Alpha-galactosidase treatment was performed on some sections by incubation at 37°C with 4 mU galactosidase in 50 mM citrate-phosphate buffer pH 4.6 for a total of 18 hours with a renewal after 6 hours. Control sections were incubated in parallel in the same buffer without the enzyme. Following treatments, rNB2 VLPs (1 µg/ml) were added for 1 hour at room temperature and the detection of binding was performed as described above.

### Hemagglutination assay

Bovine blood samples were obtained from the National Veterinary School of Nantes and human blood samples were provided by ABO phenotyped volunteer donors at INSERM U892 (Nantes, France). After collection, whole blood samples were stored at 4°C. Red Blood Cells (RBC) were packed in PBS pH 7.2 (without Ca^2+^) by centrifugation for 5 minutes at 2500 rpm. The hemagglutination activity (HA) of rNB2 VLPs and GII.4 VLPs was tested in microtitration plates with V bottomed wells (Nunc, Roskilde, Denmark). Equal volumes (25 µl) of VLPs (2.5 µg/ml) serially diluted in PBS and 1% packed RBCs in PBS were mixed and the plates were incubated for 1 hour at either room temperature or 4°C. The HA titer was the reciprocal of the greatest VLPs dilution that did allow sedimentation of the RBCs compared to negative control wells that contained buffer only.

### Purification of human natural anti-αGal antibodies

The synthetic trisaccharide Galα3Galβ4GlcNAc covalently linked to Sepharose® FF (Fast Flow 6B, Pharmacia Biotech) was obtained from Lectinity (Moscow, Russia). A chromatography column (Biorad Richmond, CA) was packed with 15 ml of immunoadsorbent and rinsed with 250 ml PBS. Thirty ml pooled human plasma were then passed through the column at a 1 ml/h flow rate. After extensive washing with PBS, bound antibodies were eluted with 20 ml of CH3COOH 0.58% in NaCl 0.9%, pH 2.8. The eluate was immediately neutralized with 20 ml of 100 mM Tris/HCl, pH 8.8 and dialyzed against PBS. The reactivity and specificity of the purified antibodies was then controlled by ELISA on coated PAA neoglycoconjugates.

### ELISA-based carbohydrate and saliva microtiter plate assays

Oligosaccharides as PAA and HSA conjugates were coated at 10 µg/ml or serially diluted onto NUNC Maxisorp immunoplates in 100 mM carbonate buffer pH 9.6 by overnight incubation at 37°C in a wet atmosphere. After blocking with 5% defatted dried cow's milk in PBS for 1 hour, VLPs (4.6 µg/ml) in PBS 5% milk were added. After incubation for 2 hours at 4°C in the case of oligosaccharides-PAA or at 37°C for oligosaccharides-HSA, rabbit anti rNB2 VLPs serum at 1/1000 dilution in PBS 5% milk was added and incubated for 1 hour at 4°C. Then, peroxidase anti-rabbit IgG (Uptima) at a 1/2000 dilution in PBS 5% milk were added and incubated for 1 hour at 4°C. Between each step, the plates were washed 3 times with PBS 5% Tween 20. The enzyme signals were detected using TMB (3, 3′, 5, 5′ tetramethylbenzidine) as substrate (BD Bioscience, San Jose, CA) and then read at 450 nm.

Saliva samples were collected from 16 human individuals of known ABO and secretor phenotypes [37], and from 16 cows from the National Veterinary School of Nantes, respectively. After collection, samples were boiled for 10 minutes and centrifuged for 5 minutes at 13,000 g. To assay rNB2 VLPs binding to saliva, microplates were coated with either human or bovine saliva diluted 1/1000 in 100 mM carbonate buffer, pH 9.6 and the assay was performed as described above using rNB2 VLPs at a 1 µg/ml concentration and a 1 h incubation at 37°C.

To detect HBGAs in bovine saliva, after coating, a blocking step was performed with ELISA Synblock reagent (Serotec, Kidlington, UK) for 2 hour at 37°C. Either peroxidase-conjugated UEA-I lectin at 2 µg/ml, monoclonal antibodies at a 1/1000 dilution, or purified human natural anti-αGal antibodies at 50 µg/ml were then added to the wells. Reagents incubations were performed at 37°C for 1 h. Binding was detected either immediately following PBS washings for the UEA-I lectin, or following a 1 h incubation at 37°C with either anti-mouse or human anti-IgG peroxidase conjugates (Uptima), at a 1/2000 dilution.

Inhibition of binding of rNB2-VLPs by human natural anti-αGal was performed by mixing VLPs at 1 µg/ml with the purified antibodies at 50 µg/ml for 2 h at 37°C prior to incubation onto the coated bovine saliva samples. After a 1 h incubation at 37°C, binding of the rNB2-VLPs was detected as above.

### NMR experiments

NMR samples contained 0.24 µg/µl or 22.5 nM VLPs in 23 mM phosphate buffer pH 7, 154 mM sodium chloride. Assuming a number of 180 monomers and 90 binding sites per capsid, this corresponds to a 4.06 µM concentration of monomers, and to a 2.03 µM concentration of binding sites. Samples contained 0.5 mM carbohydrate ligand, resulting in a ∼1∶250 molar ratio of binding sites to ligand. All experiments were carried out on a Bruker Avance 500 MHz NMR spectrometer equipped with a TCI cryogenic probe. The temperature was set to 282 K. STD NMR experiments [31] were recorded with a 3-9-19 watergate sequence and an inter-scan delay of 25 s [60]. On- and off- resonance frequencies were set at −4 and 300 ppm, respectively [32]. A train of Gaussian pulses with a pulse length of 49 ms, an inter-pulse delay of 1 ms, and an attenuation level of 50 dB was applied for selective saturation of the protein. Spectra of Galα3GalαOMe were recorded with increasing saturation times from 0.35 to 4 s, and a total of 64 to 1k scans. The resulting STD build-up curves were subjected to non-linear fitting to a mono exponential equation: 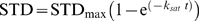 with STD being the STD signal intensity at saturation time *t*, STD_max_ being the maximal STD intensity at infinite saturation times, and *k_sat_* being the observed saturation rate constant [61]. The curve fitting was done with Origin (Microcal) and yielded the relative binding epitope [62]. STD spectra of the B antigen were recorded at only one saturation time of 2 s with 816 scans.

### Cell culture and transfection

The complete coding sequence of the *Ggta1* gene encoding the rat α1,3galactosyltransferase was cloned as previously described [21] and inserted into the PCR3.1 eukaryotic expression vector (InVitrogen, Paisley, UK). Human Embryonic Kidney (HEK) 293 cells, maintained in D-MEM/F-12 supplemented with 10% (v/v) fetal calf serum (FCS) 2 mM L-glutamine, free nucleotides (10 µg/mL), 100 U/ml penicillin and 100 µg/ml streptomycin (Gibco, Paisley, UK) were transfected with the rat Ggta1 using lipofectAMIN™ (InVitrogen) according to the manufacturer's instructions. Stable transfectants were obtained by selection with 0.5 mg/ml G418 (Gibco). Cells were then cultured in the presence of 0.1 mg/ml G418, passaged at confluence after dispersal with 0.025% trypsin in 0.02% EDTA and routinely checked for mycoplasma contamination by Hoechst 33258 (Sigma) labeling.

Porcine aortic endothelial cells (PAEC) from a wild type pig, and from a *Ggta1* knockout pig, were obtained from Dr. B. Charreau (INSERM U643, ITERT, Nantes, France). The cells were cultured in RPMI supplemented with 10% (v/v) FCS, 2 mM L-glutamine, 100 U/ml penicillin and 100 µg/ml streptomycin (Gibco) at 37°C in a 5% CO2 humid atmosphere. They were passaged at near confluence as described above.

### Flow cytometry analysis

Cells at near confluence were detached by a brief 0.025% trypsin/0.02% EDTA treatment. Viable cells, 2×10^5^ per well of 96 culture microtiter plates were incubated with either FITC-labelled GS1-B4 lectin at 10 µg/ml in PBS 0.1% gelatin or with rNB2 VLPs at 4.6 µg/ml in PBS 1% BSA for 45 min at 4°C. In the former case fluorescence analysis was performed immediately following 3 washes. In the latter case, after 3 washes, incubation was performed with the rabbit anti-NB2 serum at a 1/1000 dilution for 30 min. Following 3 washings, a 30 min incubation was then performed with FITC-labeled anti-rabbit IgG (Sigma) diluted 1/500. Finally after 3 more washings, fluorescence analysis was performed on a FACSCalibur (Becton Dickinson, Heidelberg, Germany) using the CELLQUEST program.
